# Correction:Serum Uric Acid and Mortality Form Cardiovascular Disease:EPOCH-JAPAN Study

**DOI:** 10.5551/jat.Er31591

**Published:** 2016-12-01

**Authors:** Wen Zhang, Hiroyasu Iso, Yoshitaka Murakami, Katsuyuki Miura, Masato Nagai, Daisuke Sugiyama, Hirotsugu Ueshima, Tomonori Okamura, EPOCH-JAPAN GROUP

In this article ‘ Serum Uric Acid and Mortality Form Cardiovascular Disease: EPOCH-JAPAN Study’ by Wen Zhang, et al., which appeared in JAT 2016; 23: 692-703 are incorrect.

